# Research on oil boom performance based on Smoothed Particle Hydrodynamics method

**DOI:** 10.1371/journal.pone.0289276

**Published:** 2023-07-27

**Authors:** Jiaqi Liu, Peigang Jiao, Yuntao Xu

**Affiliations:** School of Construction Machinery, Shandong Jiaotong University, Jinan, Shandong Province, China; Tongji University, CHINA

## Abstract

To address the issues of fluid-solid coupling, instability in the liquid two-phase flow, poor computational efficiency, treating the free surface as a slip wall, and neglecting the movement of oil booms in simulating oil spill containment, this study adopts the Smoothed Particle Hydrodynamics (SPH) method to establish a numerical model for solid-liquid coupling and liquid two-phase flow, specifically designed for oil boom containment and control. The DualSPHysics solver is employed for numerical simulations, incorporating optimized SPH techniques and eight different skirt configurations of the oil boom into the numerical model of two-phase liquid interaction. By setting relevant parameters in the SPH code to enhance computational efficiency, the variations in centroid, undulation, and stability of undulation velocity for different oil boom shapes are observed. The experimental results demonstrate that the improved oil boom exhibits superior oil containment performance. These findings provide a theoretical basis for the design of oil boom skirt structures.

## Introduction

Oil booms are widely acknowledged as a critical measure in emergency response to oil spills, with high practical value. They are effective in containing the spread of oil spills, thereby preventing further pollution of the marine environment [1, 2].

In order to enhance the effectiveness of oil booms as a critical measure for emergency response to oil spills and to address different scenarios, the Smoothed Particle Hydrodynamics (SPH) [3–5] numerical simulation method can be utilized to accurately simulate the undulation process and lift force of oil booms with various shapes. The undulation behavior of oil booms, particularly significant undulations, can lead to reduced oil containment effectiveness and potential failure. The findings of this research provide a scientific basis for the design of oil booms. The SPH method discretizes a continuous medium into a set of particles, and the discrete Navier-Stokes equations are locally integrated at each particle’s position based on the physical properties of the surrounding particles [6, 7]. The set of neighboring particles is determined by a distance-based function, which can be two-dimensional or three-dimensional, with the characteristic length or smoothing length denoted as h [8, 9]. In each time step, new physical quantities are calculated for each particle, and they are then updated to determine the movement of the particles [10]. Based on previous experimental and numerical research, it has been consistently shown that an optimized skirt structure of the oil boom significantly improves its effectiveness in retaining oil and prolongs its failure time. Building upon these findings, the present study further investigates the influence of the skirt structure on critical parameters such as the center, speed, and amplitude of oil boom undulation, providing additional verification of the relationship between skirt structure and oil boom performance [11].

Based on the results of previous experiments, the feasibility of the numerical model has been demonstrated. In this study, a fluid-solid interaction model was established, focusing on the undulation of oil booms under the influence of waves [12]. To achieve singularity of variables, a coupled model of Smoothed Particle Hydrodynamics (SPH) and Chrono was designed to restrict the motion of oil booms in the X and Y axes, thus providing a more accurate simulation of the undulation effect of oil booms [11, 13]. In order to realistically reflect turbulence phenomena, an improved Sub-Particle Scale (SPS) turbulence model was adopted, which has higher accuracy and reliability in predicting vortex structures and flow characteristics [14, 15]. The skirt structure of the oil boom was designed, and the undulation data of different skirt structures under waves were obtained, providing effective theoretical support for oil boom structure design [16].

The remaining sections of this article are organized as follows. Firstly, the application of the Smoothed Particle Hydrodynamics (SPH) method is introduced, including its coupling with Project Chrono and the implementation of relevant formulas. Secondly, the structural design of the oil boom is elaborated, and the numerical model is established. Subsequently, the numerical results are analyzed, and their consistency with the experimental results is examined, including simulation values for geometric center change, floating change, and speed change. Finally, the conclusion section provides a comprehensive summary of the entire article.

## Method application

### Coupling of SPH with CHRONO

**Fig 1:**
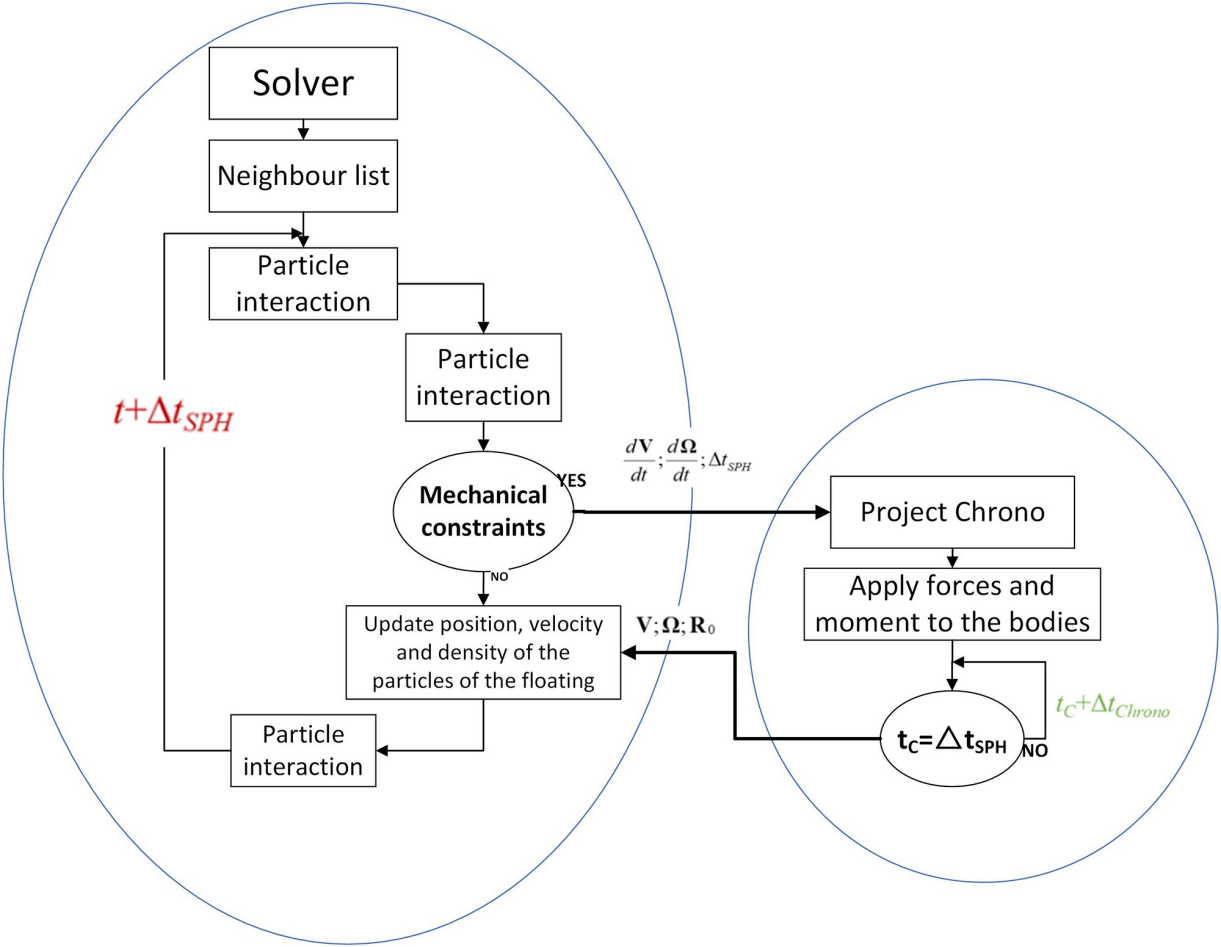
Flow chart of the coupling between SPH and Project Chrono.

The multibody solver used, Project Chrono [17, 18], is an open-source high-performance library and set of applications that enables the implementation of various applications, such as wheeled and tracked vehicles running on deformable terrain, robots, electromechanical systems, and compliant mechanisms. The solid system in CHRONO can create rigid and flexible/compatible components with constraints, motors, and contacts; components can have three-dimensional shapes for collision detection. Currently, DualSPHysics incorporates the rigid body, constraints, and collision detection components from the library. This integration allows users to describe a set of objects using meshes, specify constraints applied to the objects (selected from a list of implemented joints, hinges, and springs), and compute interactions between the objects. This approach is similar to DEM implementation but offers improved stability. Additionally, the implementation now supports realistic frictional behavior by utilizing a complete Coulomb sliding/sticking/rolling model. The implementation strategy involves coupling the two models through a message-passing interface. Once DualSPHysics calculates the quantities of the rigid bodies, time step, linear accelerations, and angular accelerations of each body (computed solely from fluid-body interactions), they are sent to our Project Chrono module. For that time step, Chrono returns linear velocities, angular velocities, and CC positions, calculated by combining the dynamics or kinematic constraints (including collisions) of the fluid set with the system [19, 20]. The coupling process between SPH and chrono is shown in Fig 1.

### SPH approximation equations

Using interpolation functions, integral equations are used to transform the conservation laws of continuous fluid dynamics from their partial differential equation form into a form suitable for particle-based simulations, providing estimated values at specific points [21]. Typically, these interpolation or weighting functions are referred to as kernel functions (*W*), and the basic principle is to approximate any function F(*r*) using integral interpolation [22].

$$\begin{array}{c} F\left(r\right)=\int F(r^{\prime })W(r-r^{\prime },h)dr^{\prime }. \end{array}$$ (1)

In the discrete representation, the interpolation approximation at point a is as follows:

$$\begin{array}{c} F(r_{a})\equiv \sum_{b}F(r_{b})\frac{m_{b}}{\rho_{b}}W(r_{a}-r_{b},h). \end{array}$$ (2)

Where h represents the smoothing length, *m*_*b*_ denotes the mass of particle b, *rho*_*b*_ represents the density of particle b such that the volume of the particle is given by *V*_*b*_ = *m*_*b*_/*ρ*_*b*_, The position vector is denoted as r = *r*_*a*_, *W*_*ab*_ = *W*(*r*_*a*_ − *r*_*b*_, *h*) is the weighting function or kernel function that represents the interaction between particles a and b.

**Fig 2:**
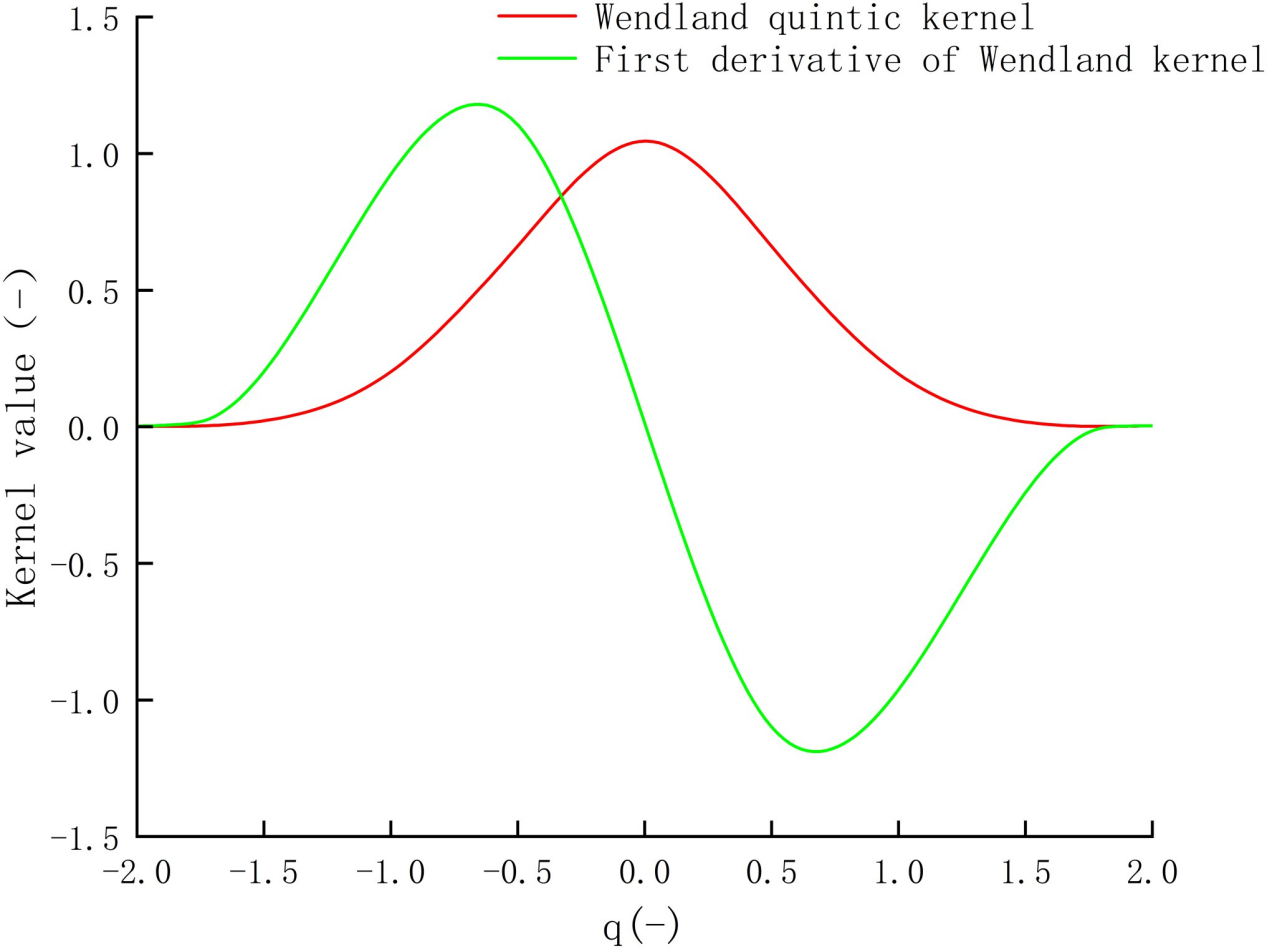
Wendland kernel function and its first derivative as a function.

The smoothing kernel must satisfy several properties, including normalization, non-negativity, decay, compact support, Dirac function condition, symmetry, and smoothness [23]. In the SPH method, the choice of the kernel function has a significant impact on the accuracy of simulation results and computational efficiency. The kernel is typically represented as a dimensionless function of the distance (q) between particles [24], where q = r/h, and r is the distance between two given particles a and b, and h (the smoothing length) controls the size of the area around particle a that includes neighboring particles. In this project, the Wendland function, as shown in Fig 2, is adopted as the kernel function, which has better smoothness and differentiability [25], making it more effective in handling problems with high fluid viscosity and significant changes in liquid surface morphology.

$$\begin{array}{c} W\left(r,h\right)=\alpha_{D}(1-\frac{q}{2}{)}^{4}\left(2q+1\right)\;0\leq q\leq 2. \end{array}$$ (3)

where *alpha*_*D*_ is defined as 7/4*πh*^2^ in two dimensions and 21/*πh*^3^ in three dimensions. In this paper, only the kernel within a radius of 2h is considered.

The Wendland kernel function and its first derivative are shown in Fig 2.

### Control equations

Gotoh et al. [26] first described the sub-particle scale (SPS) to represent the influence of turbulence in the MPS model. The SPS method involves adding a certain number of SPS particles around each SPH particle to model the shear flow in the flow field [27]. The motion equations of these SPS particles consist of a set of new equations, which include additional tension and shear viscosity terms. These terms can increase the viscosity and dissipation in the simulation, making the SPH simulation more accurate. Momentum conservation equation:

$$\begin{array}{c} \frac{dv}{dt}=-\frac{1}{\rho }\nabla P+g+v_{0}\nabla^{2}v+\frac{1}{\rho }\nabla \cdot \vec{\tau }. \end{array}$$ (4)

Where *ρ* represents fluid density, ***v*** represents the Reynolds-averaged velocity vector, P represents the Reynolds-averaged pressure, *v*_0_ represents the kinematic viscosity, ***g*** represents gravitational acceleration, $\vec{\tau }$ represents the SPS stress tensor. Laminar flow can be represented using Eq (5) as follows:

$$\begin{array}{c} (v_{0}\nabla^{2}v{)}_{a}=\sum_{b}m_{b}(\frac{4v_{0}r_{ab}\cdot \nabla_{a}W_{ab}}{\left(\rho_{a}+\rho_{b})(r_{ab}^{2}+\eta^{2}\right)})v_{ab}. \end{array}$$ (5)

Where *r*_*ab*_ = *r*_*a*_-*r*_*b*_, *v*_*ab*_ = *v*_*a*_-*v*_*b*_, *η*^2^ = 0.01*h*^2^.

In the Smoothed Particle Hydrodynamics (SPH) discretization, it is represented as:

$$\begin{array}{cc} & \frac{dv_{a}}{dt}=-\sum_{b}m_{b}(\frac{P_{b}+P_{a}}{\rho_{b}\cdot \rho_{a}})\nabla_{a}W_{ab}+g+\sum_{b}m_{b}(\frac{4v_{0}r_{ab}\cdot \nabla_{a}W_{ab}}{\left(\rho_{a}+\rho_{b})(r_{ab}^{2}+\eta^{2}\right)})v_{ab}. \end{array}$$ (6)

Where *P*_*a*_ and *P*_*b*_ represent the pressure at the respective particles a and b in the Smoothed Particle Hydrodynamics (SPH) discretization.

The vorticity viscosity hypothesis is commonly used to model the SPS (Sub-Particle Scale) stress tensor in Smoothed Particle Hydrodynamics (SPH) simulations, employing Favre averaging for compressible fluids and Einstein notation for the direction of shear stress components i and j.

$$\begin{array}{c} \frac{{\vec{\tau }}_{ij}}{\rho }=v_{t}(2S_{ij}-\frac{2}{3}k\delta_{ij})-\frac{2}{3}C_{I}\Delta^{2}\delta_{ij}|S_{ij}{|}^{2}. \end{array}$$ (7)

Where ${\vec{\tau }}_{ij}$ represents the sub-particle stress tensor, *v*_*t*_ = [(*C*_*s*_Δ*l*)]^2^|*S*|^2^ represents the turbulent vorticity viscosity, *C*_*s*_ is the Smagorinsky constant with a value of 0.12, Δ*l* is the inter-particle spacing, *k* is the turbulence kinetic energy coefficient, *C*_*I*_ is a constant with a value of 0.0066, $\left|S\right|=\sqrt{2S_{ij}S_{ij}}$, *S*_*ij*_ is the sub-particle strain tensor.

Eq (1) can be written as:

$$\begin{array}{c} \begin{array}{cc} \frac{dv_{a}}{dt}= & -\sum_{b}m_{b}(\frac{P_{b}+P_{a}}{\rho_{b}\cdot \rho_{a}})\nabla_{a}W_{ab}+g\\ & +\sum_{b}m_{b}(\frac{4v_{0}r_{ab}\cdot \nabla_{a}W_{ab}}{\left(\rho_{a}+\rho_{b})(r_{ab}^{2}+\eta^{2}\right)})v_{ab}\\ & +\sum_{b}m_{b}(\frac{{\vec{\tau }}_{ij}^{b}}{\rho_{b}^{2}}+\frac{{\vec{\tau }}_{ij}^{a}}{\rho_{a}^{2}})\nabla_{a}W_{ab}\; \end{array} \end{array}$$ (8)

The mass of each particle remains constant in this study, and only the associated density fluctuates. These density changes are calculated by solving the mass conservation equation or continuity equation in SPH form.

$$\begin{array}{c} \frac{d\rho_{a}}{dt}=\rho_{a}\sum_{b}\frac{m_{b}}{\rho_{b}}v_{ab}\cdot \nabla_{a}W_{ab} \end{array}$$ (9)

Following the approach proposed by Monaghan [28] in 1994, the fluid is treated as a weakly compressible fluid in SPH form, and the fluid pressure is determined using the equation of state.

$$\begin{array}{c} P=B[(\frac{\rho }{\rho_{0}}{)}^{\gamma }-1] \end{array}$$ (10)

Where *γ* = 7, $B=c_{0}^{2}\rho_{0}/\gamma$, *ρ*_0_ = 1000 kg/m^3^ is the reference density, $c_{0}=c(\rho_{0})=\sqrt{\left(\partial P/\partial \rho \right)}$ is the speed of sound at the reference density.

### Symplectic position verlet scheme

The symplectic position Verlet time integrator scheme has second-order accuracy in time. It is an ideal Lagrangian scheme as it is time-reversible and symmetric, without retaining diffusive terms of geometric futures. The position Verlet format without dissipative forces is given by:

$$\begin{array}{c} \begin{array}{cc} & {\text{r}}_{a}^{n+1/2}={\text{r}}_{a}^{n}+\frac{\Delta t}{2}v_{a}^{n};\\ & {\text{v}}_{a}^{n+1}={\text{v}}_{a}^{n}+\Delta t{\text{F}}_{a}^{n+1/2},\\ & {\text{r}}_{a}^{n+1}={\text{r}}_{a}^{n+1/2}+\frac{\Delta t}{2}{\text{v}}_{a}^{n+1} \end{array} \end{array}$$ (11)

However, in the presence of viscous forces and density evolution in DualSPHysics, the velocity at the n+1/2 step is required. Therefore, a velocity Verlet half step is utilized to compute the corresponding velocity for the acceleration **F**(**r**_*n*+1/2_), position **F**(**r**_*n*+1_), and the density evolution for and, respectively. The scheme implemented in DualSPHysics can be described as follows:

$$\begin{array}{c} \begin{array}{cc} & {\text{r}}_{a}^{n+1/2}={\text{r}}_{a}^{n}+\frac{\Delta t}{2}{\text{v}}_{a}^{n}\\ & {\text{v}}_{a}^{n+1/2}={\text{v}}_{a}^{n}+\frac{\Delta t}{2}{\text{F}}_{a}^{n}\\ & {\text{v}}_{a}^{n+1}={\text{v}}_{a}^{n}+\Delta t{\text{F}}_{a}^{n+1/2}\\ & {\text{r}}_{a}^{n+1}={\text{r}}_{a}^{n}+\Delta t\frac{\left({\text{v}}_{a}^{n+1}+{\text{v}}_{a}^{n}\right)}{2} \end{array} \end{array}$$ (12)

where **r**^*n*+1/2^ is substituted to **r**^*n*+1^ in Eq 11 to eliminate dependence on **u**^*n*+1^.

Finally, the density evolution follows the half-time steps of the symplectic position Verlet scheme.

$$\begin{array}{c} \begin{array}{cc} & \rho_{a}^{n+1/2}=\rho_{a}^{n}+\frac{\Delta t}{2}R_{a}^{n}\\ & \rho_{a}^{n+1}=\rho_{a}^{n}\frac{2-\varepsilon_{a}^{n+1/2}}{2+\varepsilon_{a}^{n+1/2}} \end{array} \end{array}$$ (13)

Where $\varepsilon_{a}^{n+1/2}=-(R_{a}^{n+\frac{1}{2}}/\rho_{a}^{n+1/2})\Delta t$.

## Numerical model

### Numerical model design

**Fig 3:**
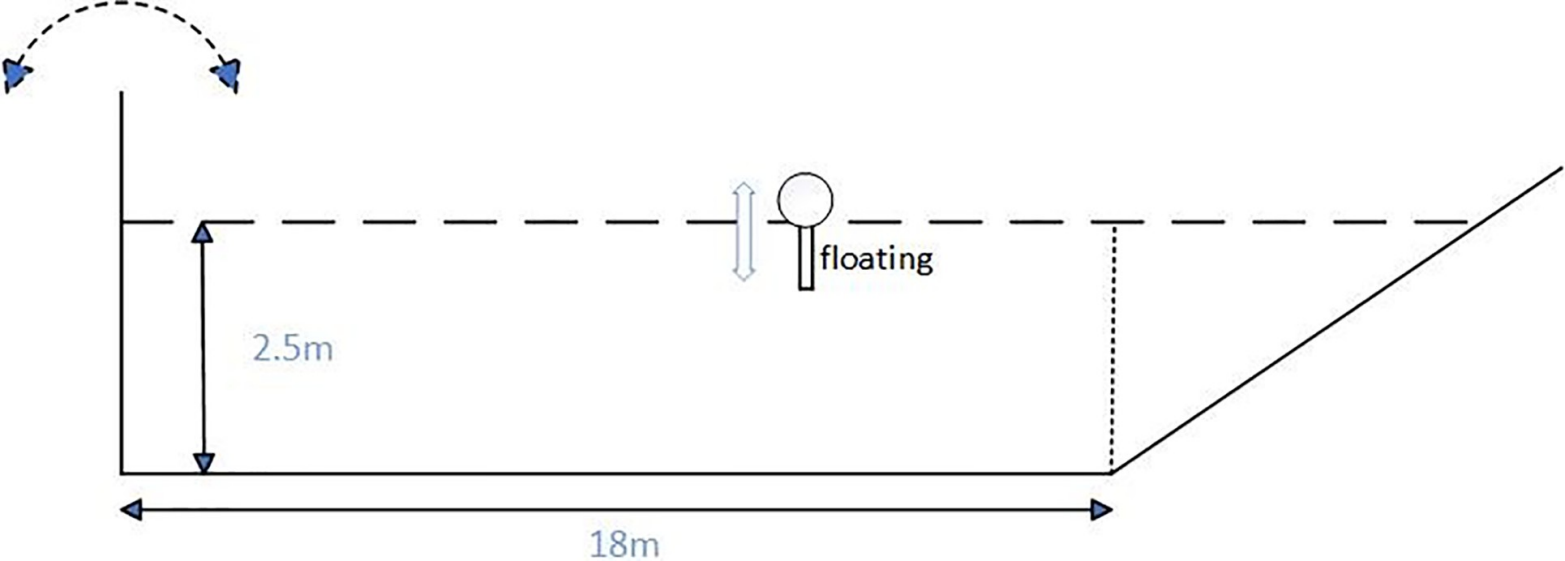
Schematic diagram of a numerical water tank.

Numerical simulations were conducted in a numerical wave tank, as shown in Fig 3. The tank had a length of 22 m and a water depth of 2.5 m. A first-order regular wave was generated by a wave maker on the left side of the tank, while a wave absorber was installed on the right side. Oil containment barriers were constrained in the X and Y axes using Project Chrono, allowing only vertical motion along the Z axis, subjected to the influence of waves and gravity.

**Fig 4:**
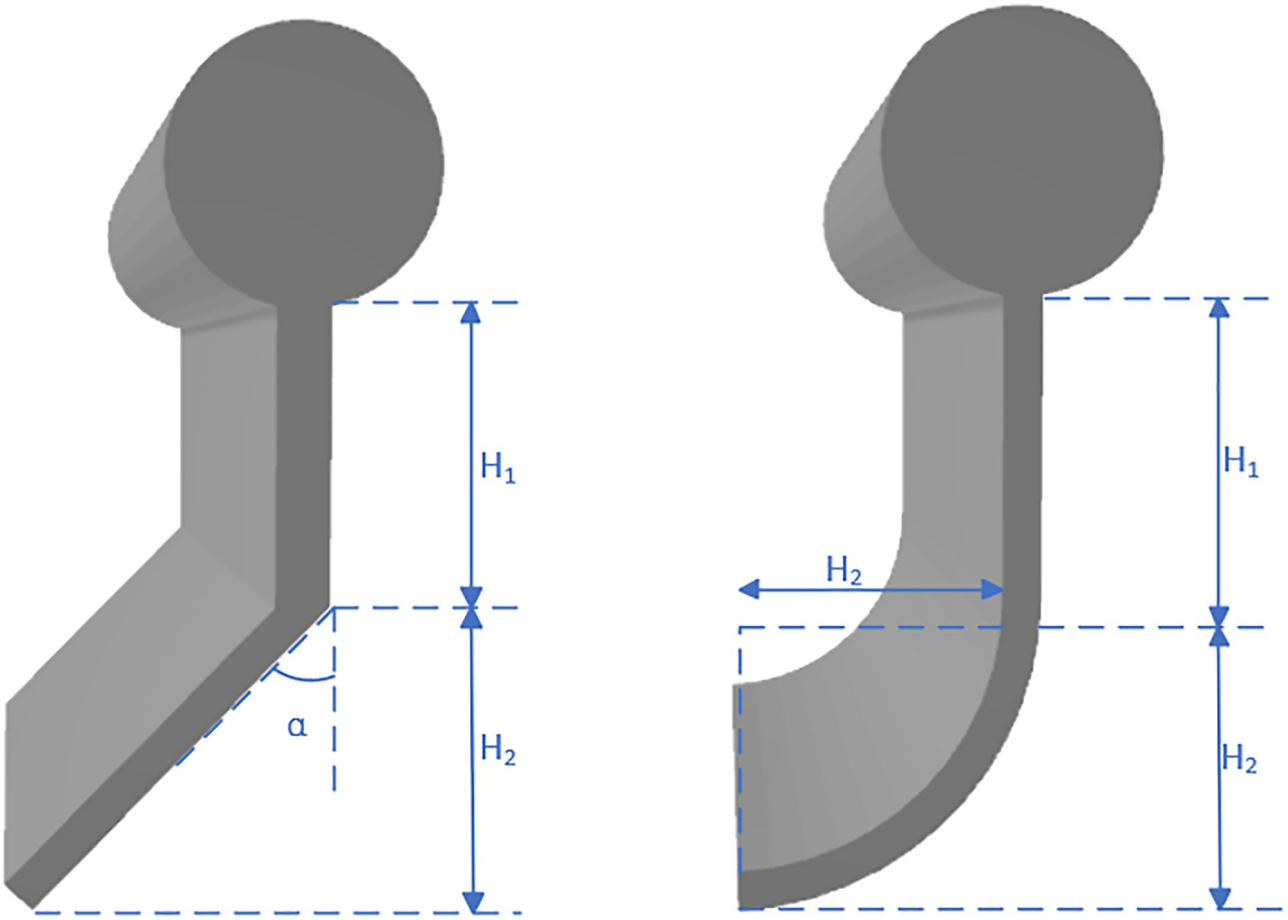
Schematic diagram of oil boom structure.

**Fig 5:**
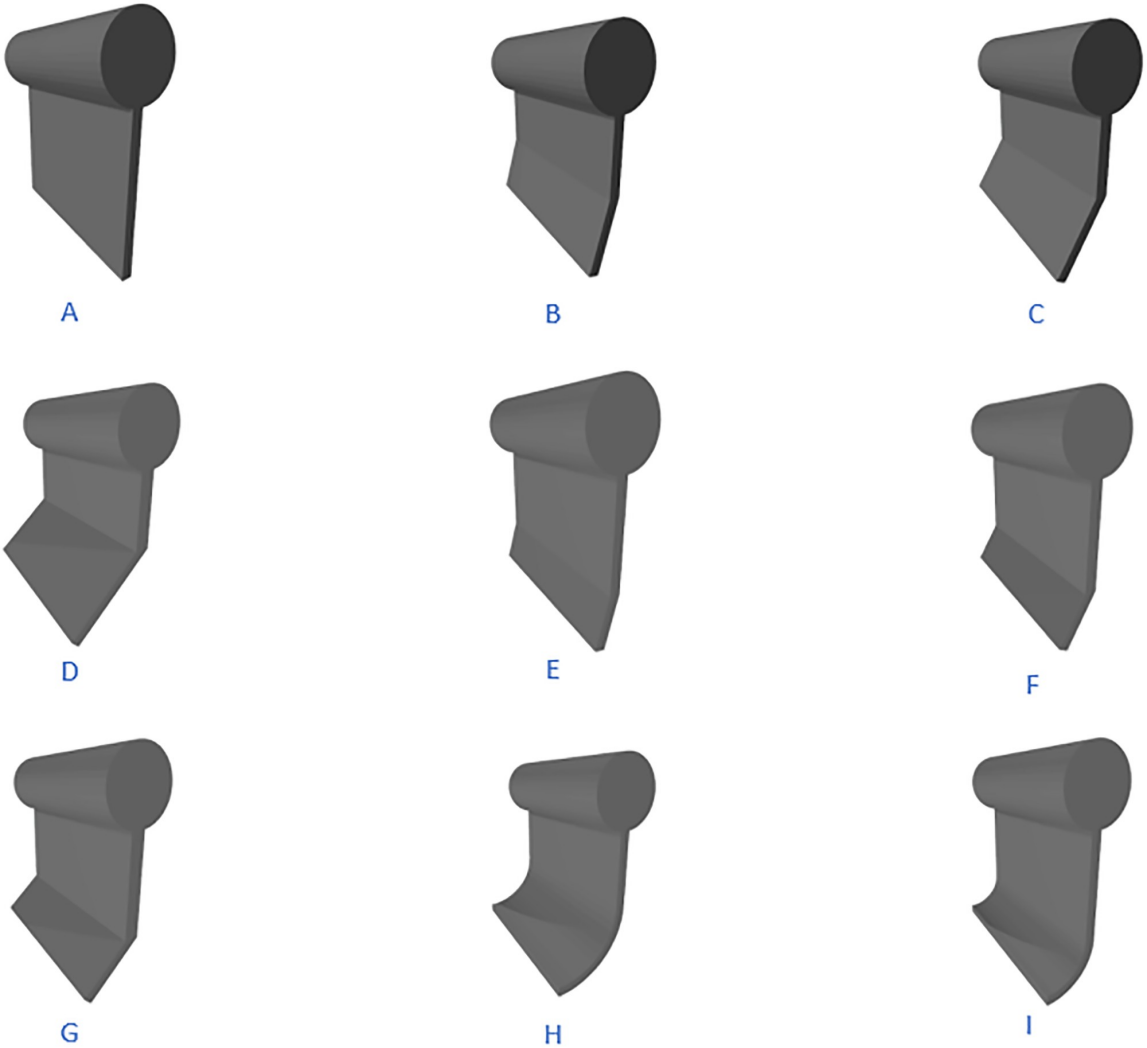
Schematic diagram of oil boom types.

In this study, eight improved skirt configurations were designed for the solid float-type oil containment barrier, as shown in Table 1 and Figs 4 and 5. The skirt height was set at 0.75 m, and the skirt angle referred to the angle between the skirt and the vertical direction.

**Table 1:** Structural parameters of oil fence.

Type	model	H1	H2	turning angle/°
I	A	0.75	0	0
	B	0.375	0.375	15
	C	0.375	0.375	30
	D	0.375	0.375	45
	E	0.5	0.25	15
II	F	0.5	0.25	30
	G	0.5	0.25	45
III	H	0.375	0.375	
	I	0.5	0.25	

The objective of this experiment is to visualize and analyze the heave motion of different skirt structures of an offshore oil boom under wave action, and investigate the effects of different skirt shapes on the heave performance of the oil boom in terms of center heave, heave velocity, heave acceleration, and heave amplitude.

### The selection of numerical parameters

The time integration scheme adopts the Symplectic method, which is second-order accurate in time and an ideal choice for Lagrangian schemes as it is time-reversible and symmetric without introducing geometric diffusion terms [29]. In this study, a Modified Dynamic Boundary Condition (mDBC) is used, where the arrangement of boundary particles is the same as in the original Dynamic Boundary Condition (DBC), and the boundary interface is located at a distance of half of the inter-particle spacing from the innermost boundary particle. Similar to the approach used by Marrone et al. [25], virtual nodes are projected into the fluid through the boundary interface for each boundary particle. The simulation parameters are listed in Table 2.

**Table 2:** Structural parameters of oil fence.

process	options
dimension	2D
domain (m)	22*4
kernel function	Wendland
time integration	Symplectic
viscosity	laminar viscosity
Turbulence	SPS
boundary	mDBC
simulation time (s)	10
initial particle distance (m)	0.02
h(m)	0.0339
fluid particles	126308
boundary particle	2136

## Analysis of numerical results

The stability of oil booms also has a significant impact on their effectiveness in controlling oil spills. Oil booms with better stability are able to maintain a relatively fixed position and exhibit smaller floating speeds under wave and flow conditions. This allows them to more effectively contain and collect floating oil on the water surface, preventing leakage or spillage. On the contrary, oil booms with poorer stability may fail to effectively resist the effects of waves and currents, resulting in oil spills bypassing the booms and reducing their effectiveness in controlling oil spills. The floating speed of oil booms directly affects their stability. When oil booms experience significant floating speeds due to wave action, their stability may be compromised, leading to reduced effectiveness in oil spill control.

### Geometric center change of oil boom

The geometric center of an oil boom is usually related to its shape, size, floating conditions, and external environmental factors. The geometric center of an oil boom refers to its centroid or geometric centroid, which is the balance point or geometric midpoint of the oil boom as a whole. The geometric center has a certain impact on oil spill containment. The position of the geometric center of an oil boom can affect its floating and movement under wave and flow conditions. If the position of the geometric center of the oil boom is stable and can maintain a relatively fixed position, it can help to surround the floating oil spill on the water surface, preventing it from leaking or overflowing, thereby improving the effectiveness of oil spill containment. However, if the position of the geometric center of the oil boom is unstable, it may experience severe floating and movement under the influence of waves and flow, which can result in the oil boom losing effective containment of the oil spill, thereby reducing the effectiveness of oil spill containment.

**Fig 6:**
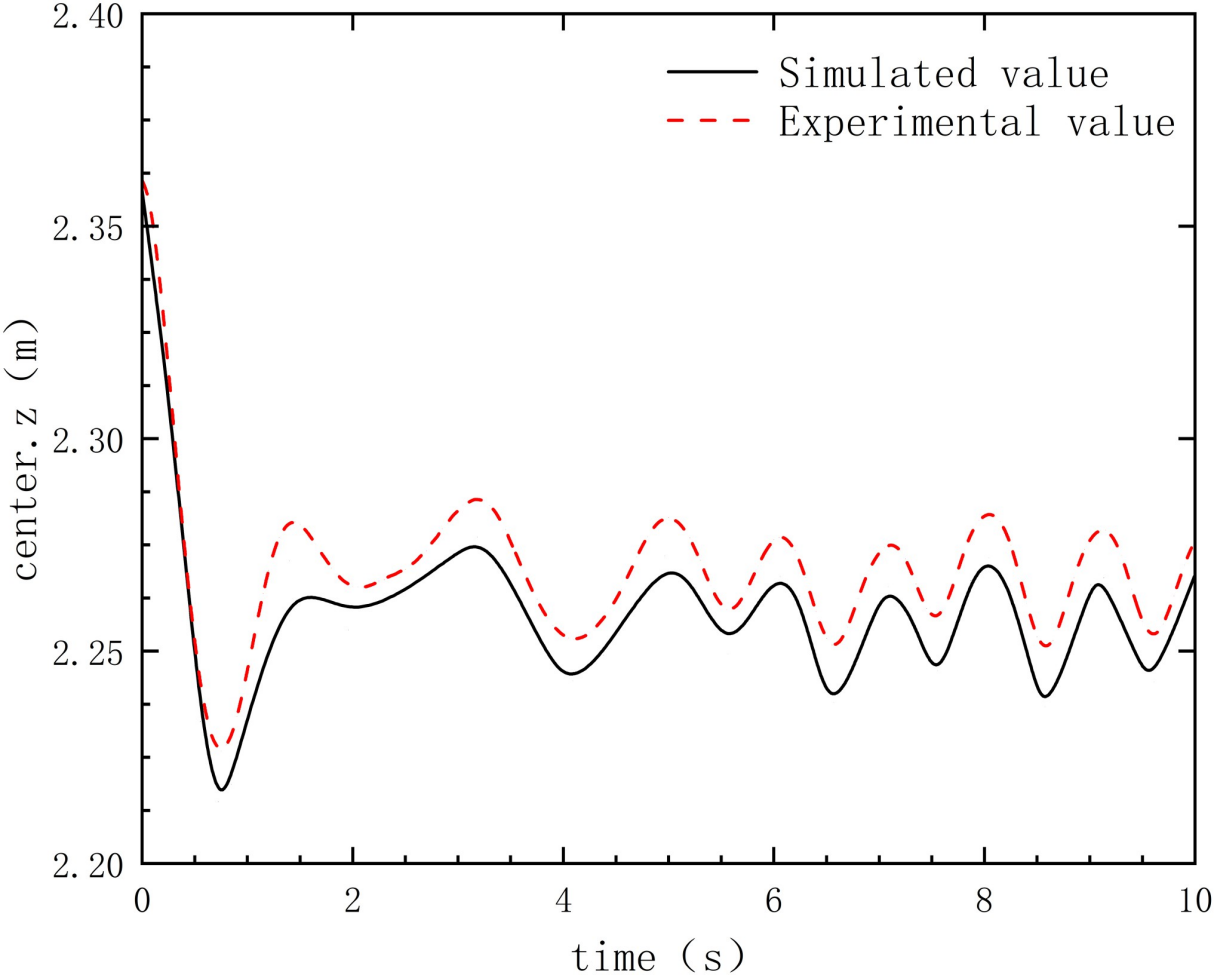
The simulated and experimental values of geometric center change.

**Fig 7:**
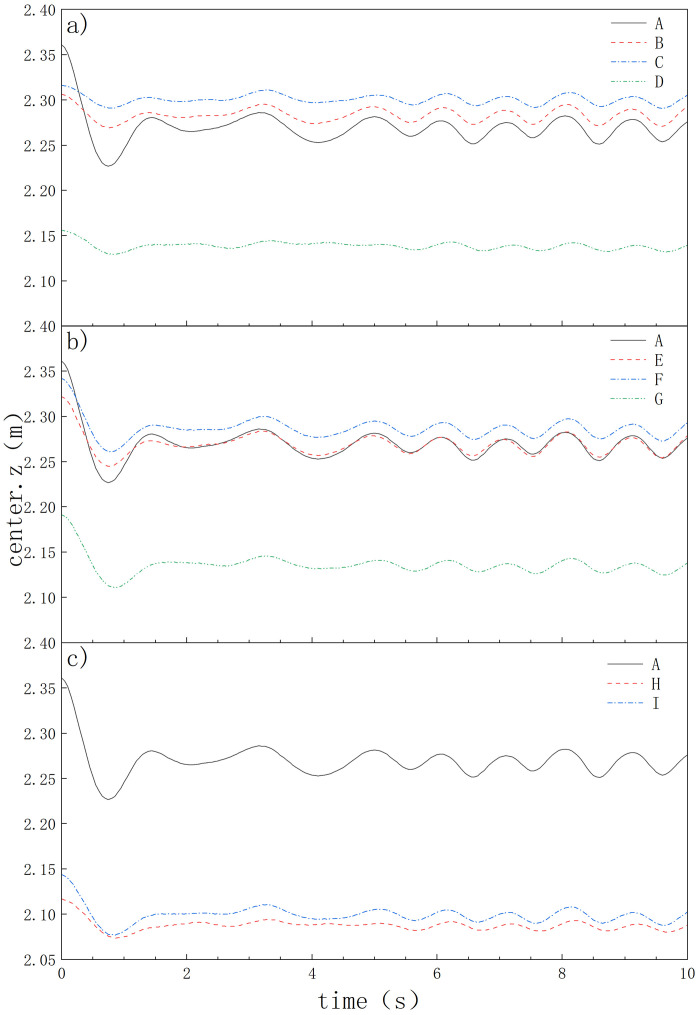
Variation diagram of the center of different oil boom geometries.

To validate the convergence property of the SPH method, five different initial particle spacings were set in this study, namely 0.05, 0.03, 0.02, 0.015, and 0.01. By ensuring both experimental accuracy and computational efficiency, an initial particle spacing of 0.02 was chosen for this study. By comparing experiments with numerical simulation experiments(Fig 6), it was found that the center variation amplitude of Type A oil boom in numerical simulation is in basic agreement with actual experimental values. This indicates that numerical simulation has high accuracy in studying the floating performance of oil booms. Numerical simulation can simulate the geometric center variation, floating amplitude, and floating speed of oil booms by simulating the same floating conditions, flow conditions, oil spill volume, wave conditions, and considering the structural design of the oil boom itself. This helps to evaluate the floating performance and stability of the oil boom, and optimize its design and structure to improve its oil spill containment effectiveness. Numerical simulation can save experimental costs and time, and improve research efficiency and accuracy. However, in order to ensure the reliability and applicability of numerical simulation results, experimental validation in actual environments is still needed. The geometric center change of the improved oil boom is shown in Fig 7.

In Table 3. The initial position of the geometric center of the oil boom is influenced differently by its own shape. The unmodified oil boom has the highest geometric center, while the geometric center of the improved oil booms is initially raised and then lowered due to the effect of the turning angle. The oil boom starts to move downward due to its own gravity, then moves upward due to buoyancy, and finally tends to dynamic equilibrium. Due to the differences in the force on the oil boom caused by the different skirt shapes, it is observed that Type A oil boom is more affected by its own gravity compared to Type I, II, and III, and its oil boom descends more, which may result in oil spilling from the top.

**Table 3:** Table of several center peak changes.

model	initial geometric center/m	lowest geometric center/m	change value/m
A	2.36085	2.22650	0.13435
B	2.30621	2.26920	0.03701
C	2.31633	2.29056	0.02577
D	2.15614	2.12918	0.02696
E	2.32172	2.24535	0.07637
F	2.34195	2.26093	0.08102
G	2.19128	2.11065	0.08063
H	2.11679	2.07366	0.04313
I	2.14362	2.07753	0.06609

### Degree of ups and downs

The floating performance of oil containment booms is not only influenced by external factors such as floating conditions, flow conditions, oil spill volume, wind and wave conditions, but also related to their own structure and design. Different structures and designs may result in different floating behaviors. The floating amplitude of oil containment booms directly affects their effectiveness in controlling oil spills. A larger floating amplitude may cause significant up-and-down movement of the boom due to wave and flow actions, resulting in a larger gap between the boom and the water surface, which may lead to oil spillage or leakage from the gap and reduce the effectiveness of oil containment. On the other hand, a smaller floating amplitude allows the boom to closely adhere to the water surface, effectively preventing oil from leaking or spilling from underneath the boom and improving the effectiveness of oil containment.

**Fig 8:**
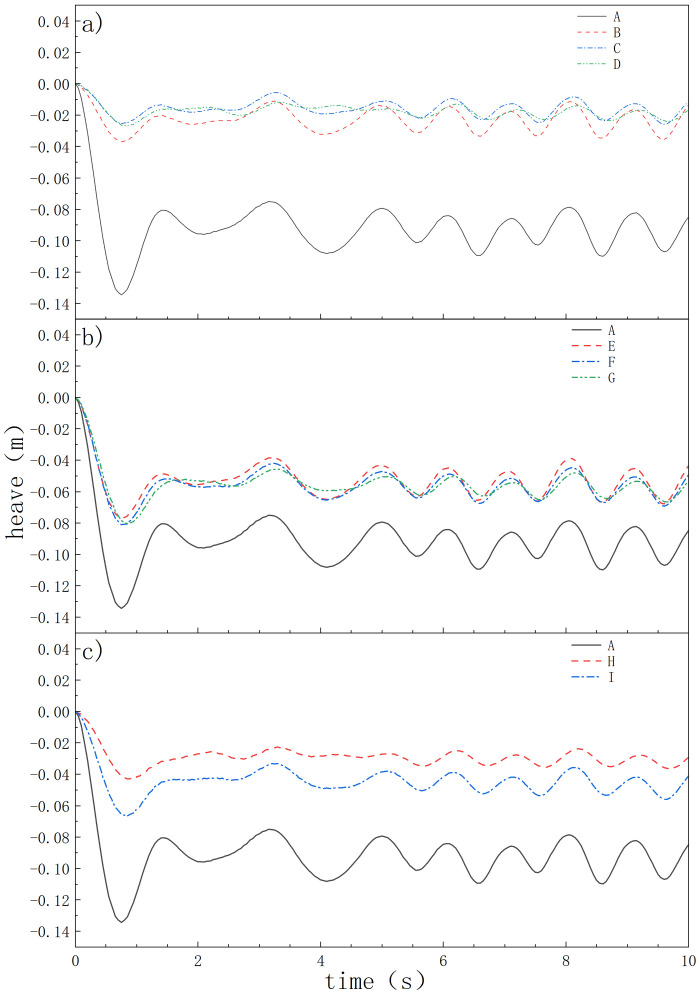
The ups and downs of oil booms of different shapes.

The degree of undulation of the oil boom is not only influenced by external factors but also by its own structure. The oil booms designed in this study have the same physical properties, such as density, mass, and immersion height, except for the skirt structure. As shown in Fig 8, the Type A oil boom has a larger initial amplitude when it enters the water, and is more affected by waves. The Type I, II, and III oil booms show significant improvements in immersion amplitude and dynamic equilibrium amplitude.

By studying the degree of undulation of the oil boom, the magnitude of its susceptibility to wave influence can be analyzed. Ignoring the undulation caused by its own gravity and taking the water surface at the water entry point as the reference plane, the interpolated undulation at dynamic equilibrium is shown in Table 4. Among the Type I, II, and III oil booms, the larger the forward angle and forward turn angle, the smaller the degree of undulation, and the less affected by waves, resulting in better oil spill prevention effects. When the forward angle is the same, compared to E, C, and G, the differences in undulation variation for B, C, and D are smaller, indicating less susceptibility to wave influence.

**Table 4:** Structural parameters of oil fence.

model	highest point/m	lowest point/m	change value/m
A	-0.07502	-0.10980	0.03478
B	-0.01136	-0.03542	0.02406
C	-0.00547	-0.02576	0.02029
D	-0.01303	-0.02397	0.01094
E	-0.03900	-0.06768	0.02868
F	-0.04207	-0.06927	0.02720
G	-0.04557	-0.06655	0.02098
H	-0.02298	-0.03635	0.01337
I	-0.03330	-0.05589	0.02259

### Stability

**Fig 9:**
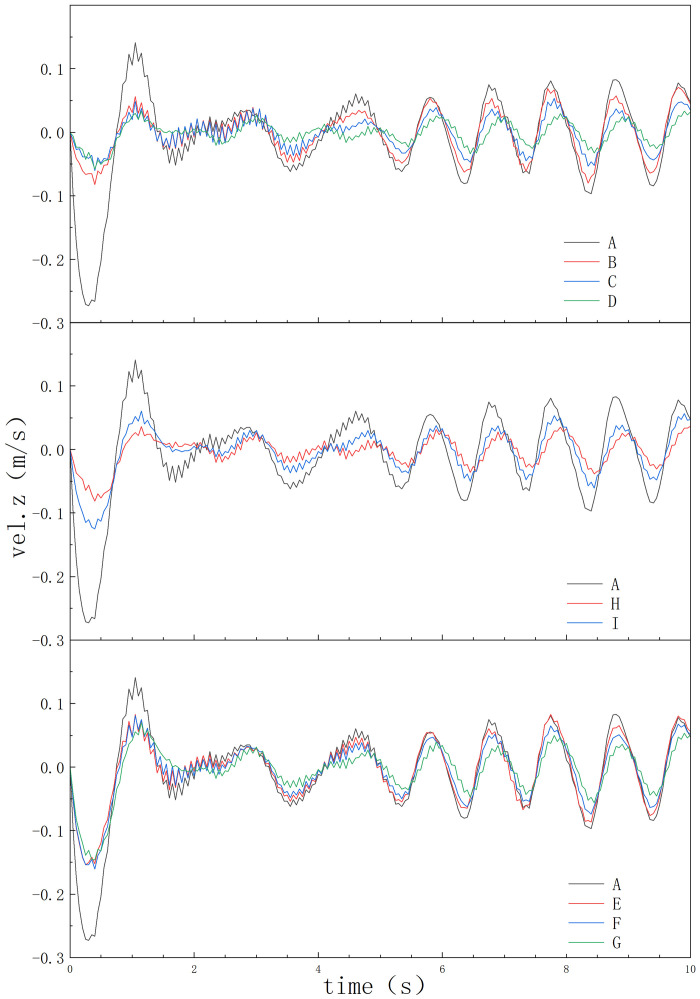
Velocity change graph of different types of oil booms.

In the same wave conditions, the speed of the oil boom is also influenced by the shape of the oil boom, as shown in Fig 9. Type I, Type II, and Type III oil booms avoid the large velocity peaks observed in Type A oil boom, improving the stability of oil boom entry into the water and its resistance to wave impacts. For Type I and Type II angle-type oil booms, the larger the angle, the stronger the stability. For Type III corner-type oil booms, the larger the corner angle, the smaller the speed, and the stronger the stability.

The floating speed and stability of oil booms are closely related to the effectiveness of oil spill control. Maintaining the stability of oil booms and controlling their floating speed can effectively enhance their ability to control oil spills, thereby reducing the impact of oil spills on the aquatic environment.

## Conclusion

This paper uses the SPH method to numerically simulate the oil spill prevention process of the improved oil boom. The process of containment and control of oil spill by the oil boom is improved, and the simulation results are more accurate. Compared with the traditional oil boom skirt structure, the improved oil containment boom skirt structure has better oil containment performance, which provides a theoretical basis for the design of the oil boom skirt skirt structure, and is compared with the experimental data to further verify the numerical simulation results accuracy. The current observations are largely qualitative, and future work will include more quantitative comparisons with laboratory experiments or field tests. More influencing factors and more types of booms used for oil spill prevention and control will be further studied. Moving forward, we will continue to optimize the SPH method by employing SPH-based differential operator formulations for the discretization of the continuity equation and momentum conservation equation [30]. Additionally, we will develop a flexible oil boom model to improve the accuracy of the simulation [31, 32].

## Supporting information

S1 Data Experimental data. (RAR)
